# Characterization of CSF2RA mutation related juvenile pulmonary alveolar proteinosis

**DOI:** 10.1186/s13023-014-0171-z

**Published:** 2014-11-26

**Authors:** Jenna Hildebrandt, Ebru Yalcin, Hans-Georg Bresser, Guzin Cinel, Monika Gappa, Alireza Haghighi, Nural Kiper, Soheila Khalilzadeh, Karl Reiter, John Sayer, Nicolaus Schwerk, Anke Sibbersen, Sabine Van Daele, Georg Nübling, Peter Lohse, Matthias Griese

**Affiliations:** Department of Pediatric Pneumology, Hauner Children’s Hospital, Ludwig-Maximilians-University, Member of the German Center for Lung Research (DZL), Munich, Germany; Department of Pediatrics, Hacettepe Üniversitesi Çocuk Göğüs Hastalıkları, Ankara, Turkey; Department of Paediatrics, Evangelisches Krankenhaus Bielefeld, Bielefeld, Germany; Children’s Hospital, Marien Hospital Wesel, Wesel, Germany; Department of Genetics, Harvard Medical School, Boston, MA USA; Pediatric Respiratory Disease Research Center, NRITLD, Shahid Beheshti University of Medical Sciences, Teheran, Iran; Institute of Genetic Medicine, Newcastle University, Newcastle upon Tyne, UK; Clinic for Paediatric Pneumonology and Neonatology, Hannover Medical School, Hannover, Germany; Department of Pediatric Pulmonology, Ghent University Hospital, Ghent, Belgium; Departments of Neurology and Palliative Care, Klinikum der Universität München, Munich, Germany; Molecular Genetics Laboratory, Institute of Laboratory Medicine and Human Genetics, Singen, Germany

## Abstract

**Background:**

Juvenile pulmonary alveolar proteinosis (PAP) due to CSF2RA mutations is a rare disorder with only a few cases described worldwide.

**Methods:**

We identified nine children with severe diffuse interstitial lung disease due to CSF2RA mutations. Clinical course, diagnostic findings and treatment were evaluated and correlated to the genotype. Functional impairment of the intracellular JAK/pStat5 signaling pathway was assessed using flow-cytometry of peripheral mononuclear cells (PBMC) and granulocytes.

**Results:**

We identified six individuals with homozygous missense/nonsense/frameshift mutations and three individuals homozygous for a deletion of the complete CSF2RA gene locus. Overall, four novel mutations (c.1125 + 1G > A, duplication exon 8, deletion exons 2–13, Xp22.3/Yp11.3) were found. Reduced STAT5 phosphorylation in PBMC and granulocytes was seen in all cases examined (n = 6). Pulmonary symptoms varied from respiratory distress to clinically silent. Early disease onset was associated with a more severe clinical phenotype (p = 0.0092). No association was seen between severity of phenotype at presentation and future clinical course or extent of genetic damage. The clinical course was favorable in all subjects undergoing whole lung lavage (WLL) treatment.

**Conclusions:**

Our cohort broadens the spectrum of knowledge about the clinical variability and genotype-phenotype correlations of juvenile PAP, and illustrates the favorable outcome of WLL treatment in severely affected patients.

**Electronic supplementary material:**

The online version of this article (doi:10.1186/s13023-014-0171-z) contains supplementary material, which is available to authorized users.

## Background

Among the interstitial lung diseases [1], pulmonary alveolar proteinosis (PAP) represents a group of disorders defined by extensive alveolar deposition of lipoproteinaceous material [2]. Several causes of PAP have been identified [3-5]. In late adolescence and adulthood, the vast majority of cases are caused by autoantibodies directed against granulocyte-macrophage colony-stimulating factor (GM-CSF). Secondary PAP develops due to impaired macrophage function from hematologic malignancies, toxic dust inhalations, and immunosuppression. In contrast, most pediatric cases of histologically diagnosed PAP can be attributed to defects in a variety of genes involved in surfactant metabolism. Mutations in the genes for surfactant protein-B (*SFTPB*), surfactant protein-C (*SFTPC*), member A3 of the ATP-binding cassette family of transporters (*ABCA3*) [6], and sometimes thyroid transcription factor 1 (*NKX2-1*) [7] lead to PAP in combination with abnormalities in the pulmonary interstitial tissue [8]. Mutations in the genes encoding the GM-CSF receptor (*CSF2RA* and *CSF2RB*), in contrast, cause pure PAP without involvement of the interstitial space. In recent years, two cases due to *CSF2RB* mutations [4,9] and 13 cases caused by *CSF2RA* gene defects have been published, including one case series [10] and single case reports [11-16]. The protein encoded by the *CSF2RA* gene is the alpha subunit of the heterodimeric receptor for colony stimulating factor 2, a member of the cytokine family of receptors that controls production, differentiation, and function of granulocytes and macrophages [17]. The *CSF2RA* gene is located in the pseudoautosomal region (PAR) of the X and Y-chromosomes.

Information on the chronic lung disease which develops in consequence of *CSF2RA* mutations is important for the management and prognosis of affected patients, but unfortunately rather scarce. In this study, we characterize the range of pulmonary phenotypes in 9 children with *CSF2RA* mutations identified and followed at our department or within the Kids Lung Register database [18]. This report includes four novel, previously unpublished mutations, and, in connection with a review of all published cases, highlights the importance of the intracellular C-terminal domain of CSF2RA for protein function.

## Methods

### Participants

The Kids Lung Register database [18] was screened for pediatric patients with pulmonary alveolar proteinosis aged 0–18 years (n = 9). All patients had been classified by experienced clinicians from 6 medical centers in 4 countries. Inclusion criteria for the study were negative GM-CSF autoantibody levels, proof of CSF2RA mutation, and exclusion of other inherited surfactant disorders. Upon inclusion in the Kids Lung Register, available follow-up data on all patients was prospectively added to the database. In this study, all follow-up data available until November 1^st^, 2013 was included. Clinical data referred to in this study always represents the patient status at admission (prior to treatment, if treatment was necessary). The study was approved by the institutional review board, the Ethikkommission der Med. Fakultät der LMU München, Pettenkoferstr. 8, 80336 Munich, Germany (EK 026–06) and all parents or guardians gave their written informed consent, and the children gave assent.

### Clinical review

Medical records were reviewed including chest radiographs, high-resolution computed tomography (HRCT) of the chest, and routine blood chemistry and hematologic tests. Some participants underwent pulmonary function testing, bronchoscopy with bronchoalveolar lavage (BAL), and consecutive examination of BAL fluid cell cytology as reported [11] or surgical biopsy as part of their clinical care. When obtained, lung biopsies were processed and evaluated using standard methods. The patients repeatedly underwent complete examinations as part of their follow-up visits. The clinical phenotype was categorized into four groups according to the patients’ worst respiratory status and physical development outcome as following: asymptomatic, mild (e.g. intermittently oxygen-dependent and/or symptomatic on exertion), moderate (constantly oxygen-dependent and/or symptomatic at rest, failure to thrive), severe (symptomatic at rest and need for endotracheal intubation and ventilation during the clinical course and/or respiratory distress syndrome, failure to thrive).

### Genetic analysis

Sequence analysis of the CSF2RA gene was performed in all participants and their parents as described previously [13]. Similarly, disorders of surfactant production involving mutations of SFTPB, SFTPC, or ABCA3 were excluded [19].

### GM-CSF-induced STAT5 phosphorylation in peripheral blood monocytes and granulocytes

GM-CSF-stimulated phosphorylation of STAT5 in peripheral blood monocytes (PBMC) and granulocytes was evaluated in six patients according to a modified protocol by Suzuki et al. [11]. Heparinized blood was processed within 24 hours. Mononuclear cells and granulocytes were isolated using a Ficoll-Paque plus gradient (GE Healthcare, Uppsala, Sweden) and Leucosep tubes (Greiner Bio One, Solingen, Germany). Cell suspensions were incubated with or without GM-CSF (Leukine, Berlex, Bayer Healthcare, Seattle, WA; 100 ng/ml, 15 minutes, 37°C). Expression of phosphorylated STAT5 (pSTAT5) was measured by flow cytometry (BD FACSCanto). In a subset of cases, cell lysates were also evaluated by Western blotting using anti-STAT5 (Santa Cruz Biotechnology; Santa Cruz, CA), anti-phospho-STAT5 (Cell Signaling), or anti-beta actin (Santa Cruz) as primary antibodies.

### Serum levels of GM-CSF and GM-CSF autoantibodies

Serum GM-CSF levels were measured in all patients by ELISA as reported [20], using commercial ELISA kits (R and D Systems), and GM-CSF autoantibody levels were determined as described [21].

### Whole lung lavage

When clinically indicated or as part of their therapeutic regimen, patients underwent whole lung lavage (WLL) therapy using an internally standardized operation procedure which has been described previously [13].

### Statistics

Numeric data were evaluated for normal distribution and variance using the Shapiro-Wilk and Levene’s test, and are presented as the mean +/− SE (parametric data) or median and minimum-maximum range (nonparametric data). Statistical comparisons were made with MS Excel 2011 and GraphPad Prism (La Jolla, CA) software, using Student’s t-test, one-way analysis of variance, Spearman’s rank correlation, point biserial correlation or Fisher’s exact test as appropriate. p values less than 0.05 were considered to indicate statistical significance.

## Results

### Participants

**Figure 1:**
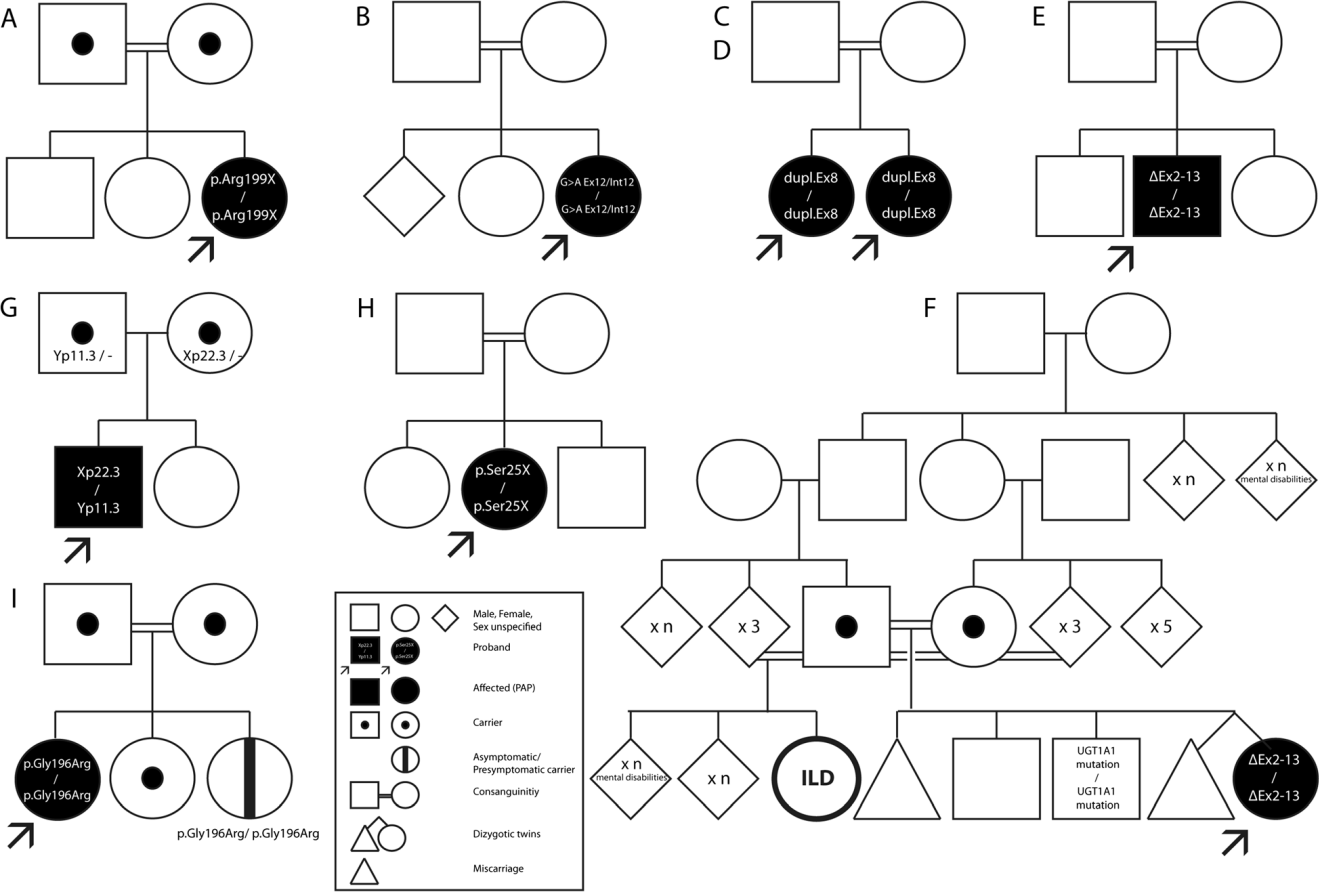
**Family pedigrees and corresponding mutations of all patients of the novel cohort.** Panels **A**. through **I**. correspond to patients **A**. through **I**. as referred to in Figure 2 and Table 2. Pedigrees of novel PAP cases. Pedigrees of all novel PAP cases are given. Consanguinity could be determined in all but one case. Of note, an uncharacterized ILD was described in a cousin of patient F. Furthermore, a homozygous but asymptomatic mutation carrier could be identified in the family of patient I.

We identified nine patients with clinical features of pulmonary alveolar proteinosis and CSF2RA mutations (Table 1). Eight patients were Caucasians and one (patient E) was Asian. Countries of origin included Belgium (n = 1), Germany with Turkish descent (n = 3), Turkey (n = 2), Iran (n = 2), and Spain with Moroccan descent (n = 1). Most parents were first-degree cousins (n =8, 89%) except for one family (patient G) (Figure 1). Patients C and D were sisters. The individual clinical details and disease courses are described in the supplementary material section (Additional file 1: Table S1).

**Table 1:** **Demographics and clinical profile of all published cases with CSF2RA mutations**

	**Current cohort (n = 9)**	**Literature (n = 11)** **[** 10-12,14,16 **]**	**Total (n = 20)**
**Family history**			
Sex (female)	7 (78%)	10 (91%)	17 (85%)
Term born	8 (89%)	9 (81%)	17 (85%)
Consanguinity	8 (89%)	≥ 3 (27%)	≥ 11 (55%)
ILD in family	1 (11%)	n.a.	≥ 1 (5%)
**Symptoms at presentation**			
Dyspnea	7 (78%)	7 (63%)	14 (70%)
Tachypnea	3 (33%)	n.a.	≥ 3 (15%)
Hypoxaemia	5 (56%)	6 (55%)	11 (55%)
Global respiratory failure	3 (33%)	4 (36%)	7 (35%)
Endotracheal intubation and ventilation	3 (33%)	n.a.	≥ 3 (15%)
Cough	5 (56%)	n.a.	≥ 5 (26%)
Fever	1 (11%)	n.a.	≥ 1 (5%)
Severe pulmonary infections prior to PAP	6 (66%)	≥ 3 (27%)	≥ 9 (45%)
Mycoplasma pneumoniae	2 (22%)	n.a.	≥ 2 (10%)
Influenza	1 (11%)	0 (0%)	1 (5%)
RSV	0 (0%)	1 (9%)	1 (5%)
**Clinical follow-up**			
Age at symptom onset, yr	3.5 (0.2-19)	4 (1.5-9)	4 (0.2-19)
Age at PAP diagnosis, yr	5.3 (2.3-19)	6 (2.5-11)	5.9 (2.3-19)
Diagnostic latency, yr	0.1 (0–5.8)	2 (1–2.5)	1 (0–5.8)
Time of follow-up, yr	2.5 (0.3-12.5)	1.7 (0.9-3)	2.5 (0–12.5)
**Outcome**			
Alive	9 (100%)	≥ 9 (82%)	18 (90%)
Best respiratory status: asymptomatic	7 (78%)	3 (27%)	10 (50%)
Last respiratory status: asymptomatic	3 (33%)	3 (27%)	6 (30%)
Disease progression: improving	7 (78%)	≥ 3 (27%)	≥ 10 (50%)
**Comorbidities**			
Failure to thrive	7 (78%)	4 (36%)	11 (55%)
PEG placement	2 (22%)	n.a.	≥ 2 (10%)
Clubbing	6 (66%)	n.a.	≥ 6 (30%)
Hepatomegaly	1 (11%)	n.a.	≥ 1 (5%)
Pectus excavatum	2 (22%)	n.a.	≥ 2 (10%)
**WLL therapy**			
WLL therapy	7 (78%)	≥ 7 (64%)	≥ 14 (70%)
Total number of WLL	19.3 (3–56)	n.a.	n.a.
Number of WLL per yr of follow-up	1.1 (0.7-6.8)	n.a.	n.a.

### Diagnostics

Lung biopsies were performed in seven patients (78%) to histologically confirm the diagnosis of PAP. In these patients, microscopic appearance showed a typical predominance of focal alveolar proteinosis with PAS-positive, granular material in the alveoli, enlarged foamy alveolar macrophages, and well-preserved alveolar walls as previously described (Additional file 2: Figure S3) [11].

Bronchoscopy was performed in every patient either as a diagnostic procedure or as part of WLL treatment. If broncho-alveolar lavage fluid (BALF) was obtained and analyzed (n = 7), milky appearance and PAS-positive stain were the common features. BALF samples of four patients were analyzed for cell differentiation, two of which showed a reduced number of macrophages in the alveolar BAL (H, I).

CT chest scans were performed in 7 cases, demonstrating a crazy paving pattern typically seen in alveolar proteinosis in all patients (Additional file 2: Figure S3).

In subjects old enough for pulmonary function testing (n = 4, patients A, F, G, H), a marked reduction of FVC was observed (FVC 16.7-67.8% predicted), and restrictive impairment was suspected.

### Genetic analysis

**Figure 2:**
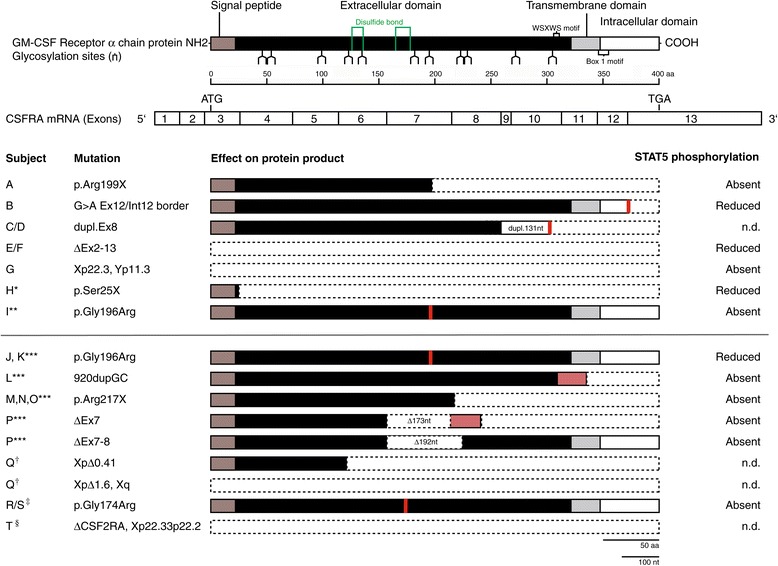
**CSF2RA mutations, resulting GM-CSF receptor α chain abnormalities, and observed impact on receptor function and clinical course.** Mutations detected in the subjects of the novel cohort (A-I) and in previously published cases (J-Q) are listed. The expected protein products are depicted with segments indicating different functional domains. Resulting deficits in STAT5 phosphorylation as determined by FACS or Western blot analysis are also provided. In addition, the clinical course is described as the initial presenting phenotype followed by the last documented condition. * [13], ** [14], *** [10], † [12], ‡ [11], § [16].

CSF2RA gene analysis was performed in all patients, revealing either missense, nonsense, point or frameshift mutations, exon duplication or extended deletions of the gene locus (Figure 2). These defects led to different extents in protein damage. In the majority of cases, there was either a whole gene deletion (n =3; patients E, F, and G) or the information of at least the C-terminal intracellular domain was lost (n =4; patients A, B, C/D, and H). In patients A and H, the transmembrane domain and parts of the extracellular domain including 4–11 disulfide bonds were also affected. Patient I, in contrast, carried a point mutation in the region of an extracellular disulfide bond.

### Clinical review

The median age at symptom onset was 3.5 (0.2 - 19) years, and the median age of diagnosis was 5.3 (2.3 to 19) years. Median time of follow-up was 2.5 (0.3-12.5) years (Table 1). There was a significant female predominance (n = 7; 78%) in this cohort. Almost all patients were term-born infants (n = 8; 89%) with no remarkable postnatal history. Concerning family history, it has to be mentioned that patient F had a cousin with an interstitial lung disease (ILD) of yet unknown cause. In all other cases, ILD could not be identified in 2nd or 3rd degree relatives (Figure 1).

Lung disease was characterized by pulmonary symptoms including chronic (tachy-)dyspnea (n = 7; 78%), hypoxemia (n = 5; 56%), and cough (n = 5; 56%) (Table 1). Global respiratory failure and need for endotracheal intubation and ventilation was present in three cases (33%). Fever and respiratory infections were not a common complication during follow-up, but six cases (67%) had severe pulmonary infections prior to diagnosis, two of which were related to Mycoplasma pneumoniae (Additional file 1: Table S1). There were no relevant comorbidities.

One prominent feature was failure to thrive in seven patients (78%). In two cases (22%), transient percutaneous endoscopic gastrostomy (PEG) tube feeding had to be applied. Median weight-for-age and length-for-age both averaged at the lower bottom of CDC growth charts [22] (Additional file 3: Figures S1 and Additional file 4: Figure S2).

All patients in this population were alive at the end of the study, with six patients showing improvement in pulmonary function. Three cases were last noted to be asymptomatic (Additional file 1: Table S1). Whole lung lavage (WLL) treatment was used in the majority of cases (n = 7; 78%). In two patients (patients H, I), periodical WLL treatment every 4–8 weeks was applied over several years to maintain a stable clinical course. The number of WLL ranged from 3–56 (median 19.3) in total or 0.7-6.8 treatments per year of follow-up.

Given the wide variety of disease severity and age at symptom onset, we investigated whether a correlation between the presenting phenotype and the age of onset was present. For this evaluation, disease severity was ranked from asymptomatic to severely affected based on physical development and worst respiratory status (also see Methods section). Of note, the severity of symptoms was inversely correlated with disease onset as reflected by the time of diagnosis (Spearman’s rank: p =0.0092).

Furthermore we investigated whether a correlation between the extent of the genetic damage and the clinical phenotype could be determined (Table 2). All patients of the current cohort as well as previously published cases were graded to one of four categories ranging from asymptomatic to severely affected as stated above. Subsequently, Fisher’s exact test was performed to determine whether the loss of the intracellular domain (exon 11) was correlated to a more severe phenotype. However, no correlation between the severity of the phenotype and the extent of genetic damage was found (p =0.18). Although in patients with exon 11 deletions a tendency towards earlier disease onset was noted (time of diagnosis: 4.9 ± 3.0 (exon 11 absent) vs. 8.1 ± 4.7 (exon 11 present) years), this observation also failed to reach statistical significance (point biserial correlation: p =0.091).

**Table 2:** **Therapy, severity of clinical phenotype and course of the disease in different CSF2RA mutations**

	**CSF2RA mutation**				
**Subject**	**Allele 1**	**Allele 2**	**Symptom onset**	**WLL**	**Course**
A	p.Arg199X	p.Arg199X	severe	yes	improving
B	G > A Ex12/Int12 border	G > A Ex12/Int12 border	moderate	yes	improving
C	dupl.Ex8	dupl.Ex8	moderate	yes	improving
D	dupl.Ex8	dupl.Ex8	asymptomatic	no	asymptomatic
E	ΔEx2-13	ΔEx2-13	moderate	yes	improving
F	ΔEx2-13	ΔEx2-13	asymptomatic	no	asymptomatic
G	Xp22.3	Yp11.3	severe	yes	improving
H	p.Ser25X	p.Ser25X	severe	yes	improving
I	p.Gly196Arg	p.Gly196Arg	severe	yes	improving
J	p.Gly196Arg	p.Gly196Arg	mild	no	improving
K	p.Gly196Arg	p.Gly196Arg	moderate	yes	stable
L	920dupGC	920dupGC	severe	yes	improving
M	p.Arg217X	p.Arg217X	asymptomatic	no	asymptomatic
N	p.Arg217X	p.Arg217X	severe	yes	n.a.
O	p.Arg217X	p.Arg217X	moderate	yes	n.a.
P	ΔEx7	ΔEx7-8	severe	yes	improving
Q	XpΔ0.41	XpΔ1.6, Xq	severe	yes	death
R	p.Gly174Arg	p.Gly174Arg	severe	yes	n.a.
S	p.Gly174Arg	p.Gly174Arg	mild	n.a.	n.a.
T	Xp22.33p22.2	ΔCSF2RA	n.a.	n.a.	n.a.

### GM-CSF receptor function analysis

All patients included in this study had negative GM-CSF autoantibody levels as measured by ELISA (data not shown). All of them also had elevated GM-CSF levels in both serum (n = 3) and, when obtained, in bronchoalveolar lavage fluid (n = 4) as compared to 1) controls, 2) adult PAP related to GM-CSF auto-antibodies, and 3) juvenile secondary PAP (data not shown).

**Figure 3:**
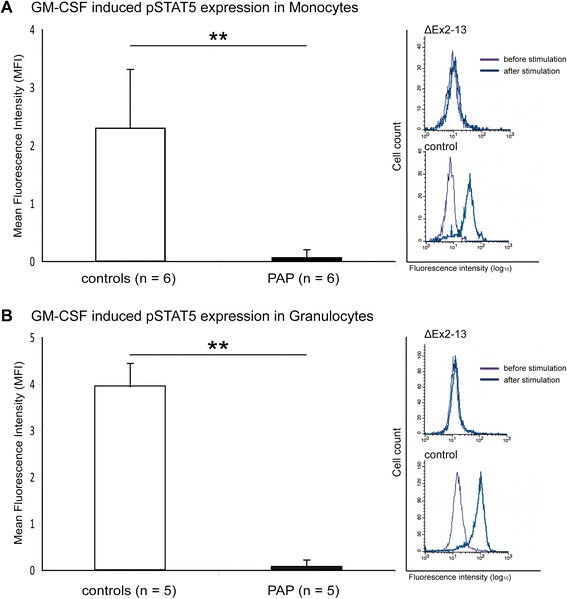
**STAT5 phosphorylation is diminished in all examined cases.** STAT5 phosphorylation was determined by FACS analysis in monocytes (n = 6) and granulocytes (n = 5) after stimulation with GM-CSF. One of the patients’ parents was used as a control in each experiment. **A**. In monocytes, almost no MFI increase is visible in PAP patients as compared to controls. The right panels demonstrate an exemplary MFI shift in a control, whereas the patient’s signal remains unchanged after stimulation. **B**. A similar pattern is seen in granulocytes.

Qualitative and quantitative analysis of the GM-CSF receptor function was performed in all but three patients (E, C, D), where no material was available. Flow cytometry of peripheral blood cells was used to investigate the signal transduction following activation of the GM-CSF receptor. Phosphorylation of STAT5 (pSTAT5) was either markedly reduced or abolished in all 6 subjects investigated (Figure 3).

## Discussion

### Genotype-phenotype relationships in our cohort and in view of published cases

In the population of PAP patients described here, we observed a wide variability regarding the clinical phenotypes and disease courses. This was true in both our population and the cases published to date.

For example, we identified two patients (A, H) with stop codon mutations which resulted in the loss of more than 50 percent of the GM-CSF receptor alpha protein (Figure 2). Both patients showed a severe clinical course, including global respiratory failure with a need for endotracheal intubation and ventilation and failure to thrive with a need for percutaneous endoscopic gastrostomy (PEG) placement, and displayed clinical signs of chronic lung disease. Furthermore, two boys (patients E, G) with whole gene deletions both developed dyspnea, cough, chronic tachypnea, crepitation, failure to thrive and, in one case, respiratory failure (E). However, it has to be noted that exon 11 loss is not necessarily always associated with a severe clinical phenotype. One female patient (F) with a homozygous deletion of CSF2RA exons 2–13 never displayed any symptoms of chronic lung disease. She has only mild obstructive lung disease according to her lung function tests.

Conversely, a severe clinical phenotype with failure to thrive and need for oxygen supplementation was observed in three patients with point mutations or small deletions in the extracellular domain (patients I, P, R). Of note, point mutations p.Gly196Arg and p.Gly174Arg were both observed in more than one case (p.Gly196Arg: patients I, J, K, p.Gly174Arg: patients R, S), and resulted in a variable phenotype, ranging from mild symptoms to severe phenotypes.

Interestingly, divergent clinical courses were also observed in siblings or non-related individuals with identical CSF2RA mutations resulting in partial or complete gene deletion (patients C/D, E/F). This phenomenon has been described before [10,14], and it implies that the phenotype is not solely determined by the CSF2RA gene alteration. In our cohort, the two sisters with exon 8 duplication (C, D) showed different clinical courses. While the younger one (C) had moderate pulmonary symptoms such as intermittent dyspnea and cough, and was diagnosed at the age of 6 years, her sister’s (D) genetic diagnosis was made by chance during the diagnostic evaluation of the family. Years later, at the age of 19 years, she developed mild chronic cough which needed no invasive treatment so far.

In summary, no correlation between the severity of the initial phenotype and the extent of genetic damage could be identified. However, patients with extensive gene deletions including the intracellular domain (exon 11) showed a tendency towards earlier disease onset. It was suggested that penetrance may be important in familial PAP [11]. Other factors contributing to symptom severity have not yet been identified, though respiratory tract infections may play a role in disease onset (Table 1) [14].

### GM-CSFR functional analysis is sensitive for mutation carriers

In the current study, all symptomatic and non-symptomatic patients with CSF2RA mutations that could be investigated (n = 6) showed a severe impairment of GM-CSF-induced phosphorylation of STAT5 in peripheral monocytes and granulocytes. Of note, no correlation between STAT5 phosphorylation impairment and the size of the gene deletions was detected. Together with findings of an impaired STAT5 signaling pathway in previously published cases [10], our data implicate that STAT5 functional analysis may be used as a sensitive tool to identify GM-CSF receptor α chain mutation carriers prior to genetic testing. Given the overall low prevalence of PAP due to CSF2RA mutations, determination of GM-CSF-levels as well as testing for GMCSF-autoantibodies should be conducted prior to STAT5 functional analysis especially in patients with a disease onset in late childhood or early adulthood. Since investigating STAT5 phosphorylation in monocytes requires relatively large blood samples, our data further suggest that granulocytes may serve as an alternative. This could be relevant if PAP is suspected in young infants.

### Course of disease and response to WLL treatment

In the present study, a huge variety of clinical PAP phenotypes was observed, ranging from clinically silent to acute respiratory failure and failure to thrive. Interestingly, the presenting phenotype at the time of diagnosis was more severe in younger patients. It has to be noted that no death as a direct consequence of PAP was observed. To date, one case report describes a patient with a severe initial phenotype who died due to complications after a bone marrow transplant (patient Q) [10,12]]. In all other cases, the clinical phenotype was at least stable during the follow-up. In most cases, patients improved over the course of disease, possibly due to individualized treatment concepts applied to each patient.

In our cohort, treatment varied from regular follow-up investigations in asymptomatic patients to frequent, periodical WLL, which were conducted over several years to either maintain a stable clinical course, or to even improve the clinical outcome. It has been repeatedly shown that WLL may lead to symptomatic relief [10,13]. For example, patient H first showed a severe clinical course including respiratory distress. After more than a decade of one- or two-monthly WLL treatment [13], disease activity slowly waned. The patient is now without WLL for more than one year and shows normal clinical and radiological findings (details see Additional file 1: Table S1).

Several lines of evidence suggest that WLL treatment may not only provide symptomatic relief, but may act in favour of a benign clinical disease course. Of note, two cases of siblings with identical GM-CSF receptor α chain mutations and proven functional receptor impairment in flow cytometry investigations have been reported where only one sibling developed PAP [14]. It was noted that in the clinically unaffected siblings no severe respiratory infections or any other causes leading to changes in their alveolar environment could be identified. It was thus concluded that certain triggers such as a severe initial pulmonary infection are required to disturb the alveolar surfactant balance and to initiate PAP. Therefore, it could be argued that repeated WLL helps establishing a novel alveolar equilibrium and may thus finally lead to complete cessation of PAP. This hypothesis is supported by findings in adult autoimmune PAP patients, where a single WLL treatment is sufficient for complete and continuous symptom relief in about 50% of the patients in spite of high auto-antibody levels [21]. In our cohort, patient H, although requiring a prolonged course of treatment, reached complete symptom relief with normal clinical and radiological findings after initially presenting with a severe phenotype. Therefore, clinical experience and theoretical implications strongly favor the repeated application of WLL therapy especially in severely affected patients.

While such individual concepts currently remain the treatment of choice, novel developments point towards advanced therapeutic options such as organotopic transplantation of marcrophage progenitors [23]. Furthermore, recent studies yielded promising results concerning patient-specific, bone-marrow derived, genetically corrected monocytes and macrophages, making individualized curative therapeutic approaches a promising perspective [15].

## Conclusions

In summary, the current study extends the clinical body of knowledge on juvenile PAP. During the prolonged clinical follow-up, a clear symptomatic benefit of repeated WLL therapy was seen in severely affected patients. Mild cases were usually self-limiting over time. While all CSF2RA mutations led to a severe impairment of the STAT5 signaling pathway, no correlation between the severity of the clinical phenotype and the extent of genetic damage was seen. However, we observed a tendency towards earlier disease onset in patients with loss of the transmembranous domain of the GM-CSF receptor α chain. Our approach to the infant and child with pulmonary alveolar proteinosis of unclear origin is to locate the defect and administrate its most effective treatment as soon as possible. STAT5 functional analysis in peripheral blood cells and subsequent genetic testing is the method of choice to classify the pathogenetic nature of this rare condition. Long-term, web-based clinical follow-up of patients with CSF2RA mutation related juvenile alveolar proteinosis in registers will be a helpful tool to further extend the body of knowledge on this rare condition, and to create ready-to-trial populations for emerging therapeutic approaches.

## Availability of supporting data

The data set supporting the results of this article is available online as Additional file 1: Table S1 at “www.ojrd.com“.

## Additional files

Additional file 1: Table S1. Individual genetic and clinical data of all patients with CSF2RA mutations reported up to now. Supplementary table with individual clinical and demographic data as well as brief case summaries of patients A-I. Additional file 2: Figure S3. Radiological and histopathological findings in juvenile PAP. A./B. Bilateral ground-glass opacifications and infiltrates as commonly seen in PAP (A. male, 9y; B. female, 5y). C. CT chest scan displaying a crazy paving pattern (female, 6y). D-F. Lung biopsy tissue shows PAS positive granular material in the alveoli. Additional file 3: Figure S1. Growth charts of female PAP patients. Stature-for-age and weight-for-age development of female PAP patients displayed on CDC growth charts [22]. Mostly, weight-for-age development fails to reach the 50th percentile. Of note, patient H showed marked improvement of physical development after initiation of regular WLL treatment at the age of 10 years. Additional file 4: Figure S2. Growth charts of male PAP patients. Stature-for-age and weight-for-age development of male PAP patients displayed in CDC growth charts [22]. In general, especially the weight-for-age parameters show a constant development below the 10th percentile, exceeding the developmental deficits seen in female patients.
