# Supplementary material for: Characterization of CSF2RA mutation related juvenile pulmonary alveolar proteinosis
Source: Orphanet J Rare Dis. 2014 Nov 26;9:171. doi: 10.1186/s13023-014-0171-z (PMC4254258; doi:10.1186/s13023-014-0171-z)
Supplement: Additional file 1: Table S1. — Individual genetic and clinical data of all patients with CSF2RA mutations reported up to now. Supplementary table with individual clinical and demographic data as well as brief case summaries of patients A-I. [file 13023_2014_171_MOESM1_ESM.docx]

**Additional file 1: Table S1: Individual genetic and clinical data of all patients with *CSF2RA* mutations reported up to now**

|  | Current cohort | | | | | | | | |  |  |
| --- | --- | --- | --- | --- | --- | --- | --- | --- | --- | --- | --- |
|  | A | B | C | D | E | F | G | H | I |  |  |
| Allele 1 | p.Arg199X | G>A Ex12/Int12 border | duplEx8 | duplEx8 | ΔEx2-13 | ΔEx2-13 | Xp22.3 | p.Ser25X | p.Gly196Arg |  |  |
| Allele 2 | p.Arg199X | G>A Ex12/Int12 border | duplEx8 | duplEx8 | ΔEx2-13 | ΔEx2-13 | Yp11.3 | p.Ser25X | p.Gly196Arg |  |  |
|  |  |  |  |  |  |  |  |  |  |  |  |
| Sex (female) | yes | yes | yes | yes | no | yes | no | yes | yes |  |  |
| Term born | no | yes | yes | yes | yes | yes | yes | yes | yes |  |  |
| Consanguinity | yes | yes | yes | yes | yes | yes | no | yes | yes |  |  |
| *Symptoms and signs* | |  |  |  |  |  |  |  |  |  |  |
| Dyspnea | yes | yes | yes | no | yes | no | yes | yes | yes |  |  |
| Hypoxaemia | yes | no | no | no | yes | no | yes | yes | yes |  |  |
| Global respiratory failure | yes | no | no | no | yes | no | no | yes | no |  |  |
| Failure to thrive | yes | yes | yes | no | yes | no | yes | yes | yes |  |  |
| Infection prior to PAP diagnosis | influenza | no | no | no | pneumonia | mycoplasma pneumonia | | acute bronchitis | mycoplasma pneumonia |  |  |
| *Clinical follow-up* | |  |  |  |  |  |  |  |  |  |  |
| Age at symptom onset, yr | 3,5 | 5,7 | 0,2 | 19 | 4,4 | 7,1 | 2,2 | 2,4 | 2,6 |  |  |
| Age at PAP diagnosis, yr | 3,6 | 5,9 | 6 | 19 | 5,3 | 10,5 | 2,3 | 2,5 | 2,7 |  |  |
| Diagnostic latency, yr | 0,1 | 0,2 | 5,8 | 0 | 0,9 | 3,4 | 0,1 | 0,1 | 0,1 |  |  |
| Time of follow up, yr | 2,2 | 2,5 | 7 | 0 | 9,8 | 1,3 | 12,3 | 12,5 | 2,5 |  |  |
| *Diagnostics and treatment* | | |  |  |  |  |  |  |  |  |  |
| Bronchoscopy and BAL | yes | yes | yes | no | yes | no | yes | yes | yes |  |  |
| Lung biopsy | yes | no | yes | no | yes | yes | yes | yes | yes |  |  |
| Lung function tests - restrictive pattern | n.a. | no | yes | no | n.a. | yes | yes | yes | no |  |  |
| WLL therapy | yes | yes | yes | no | yes | no | yes | yes | yes |  |  |
| Number of WLL per yr of follow-up | 1,8 | 1,2 | 0,7 | n.a. | n.a. | n.a. | 1,0 | 4,5 | 6,8 |  |  |
| *Outcome* |  |  |  |  |  |  |  |  |  |  |  |
| Alive | yes | yes | yes | yes | yes | yes | yes | yes | yes |  |  |
| Best respiratory status - asymptomatic | no | yes | yes | yes | no | yes | yes | yes | yes |  |  |
| Respiratory status at end of follow-up - asymptomatic | no | no | no | yes | no | yes | no | yes | no |  |  |
| Disease progression - improving | yes | yes | yes | yes | no | n.a. | no | yes | yes |  |  |
|  | Suzuki et al 2010 [10] | | | | | | | Martinez-Moczgemba et al 2008 [12]; [10] | Suzuki et al 2008 [11] | | Auger et al 2013 [16] |
|  | J | K | L | M | N | O | P | Q | R | S | T |
| Allele 1 | p.Gly196Arg | p.Gly196Arg | 920duplGC | p.Arg217X | p.Arg217X | p.Arg217X | ΔEx7 | XpΔ1.6 | p.Gly174Arg | p.Gly174Arg | Xp22.33p22.2 |
| Allele 2 | XpΔ1.6 | XpΔ1.6 | ? | p.Arg217X | p.Arg217X | p.Arg217X | ΔEx7-8 | XqHouston | Xp22.3 | Xp22.3 | ΔCSF2RA |
|  |  |  |  |  |  |  |  |  |  |  |  |
| Sex (female) | yes | yes | yes | no | yes | yes | yes | yes | yes | yes | yes |
| Term born | yes | yes | yes | yes | yes | yes | yes | n.a. | yes | yes | no |
| Consanguinity | n.a. | n.a. | n.a. | yes | yes | yes | n.a. | n.a. | n.a. | n.a. | no |
| *Symptoms and signs* | |  |  |  |  |  |  |  |  |  |  |
| Dyspnea | no | yes | yes | no | yes | yes | yes | yes | yes | no | n.a. |
| Hypoxaemia | no | no | yes | no | yes | yes | yes | yes | yes | no | n.a. |
| Global respiratory failure | no | no | yes | no | yes | no | yes | yes | no | no | n.a. |
| Failure to thrive | yes | yes | no | no | no | no | no | n.a. | yes | yes | n.a. |
| Infection prior to PAP diagnosis | n.a. | n.a. | n.a. | n.a. | pneumonia | n.a. | n.a. | RSV bronchiolitis | no | no | yes |
| *Clinical follow-up* | |  |  |  |  |  |  |  |  |  |  |
| Age at symptom onset, yr | n.a. | 4 | 8 | n.a. | 1,5 | 1,5 | 9 | n.a. | 4 | n.a. | 1,2 |
| Age at PAP diagnosis, yr | 8 | 6 | 10 | 5 | 2,5 | 4 | 11 | 3 | 6 | 8 | n.a. |
| Diagnostic latency, yr | n.a. | 2 | 2 | n.a. | 1 | 2,5 | 2 | n.a. | 2 | n.a. | n.a. |
| Time of follow up, yr | 3 | 3 | 3 | 0,9 | 1,7 | 1,3 | 1 | n.a. | n.a. | n.a. | n.a. |
| *Diagnostics and treatment* | | |  |  |  |  |  |  |  |  |  |
| Bronchoscopy and BAL | no | yes | yes | yes | yes | yes | yes | yes | yes | yes | yes |
| Lung biopsy | no | yes | yes | no | no | no | no | yes | yes | n.a. | n.a. |
| Lung function tests - restrictive pattern | no | yes | yes | n.a. | n.a. | n.a. | yes | n.a. | n.a. | n.a. | n.a. |
| WLL therapy | no | yes | yes | no | yes | yes | yes | yes | yes | n.a. | n.a. |
| Number of WLL per yr of follow-up | n.a. | 2,0 | 4,4 | n.a. | 14,2 | 11 | 16 | n.a. | n.a. | n.a. | n.a. |
| *Outcome* |  |  |  |  |  |  |  |  |  |  |  |
| Alive | yes | yes | yes | yes | yes | yes | yes | no | yes | yes | n.a. |
| Best respiratory status - asymptomatic | yes | no | no | yes | no | no | yes | n.a. | n.a. | n.a. | n.a. |
| Respiratory status at end of follow-up - asymptomatic | yes | no | no | yes | no | no | yes | n.a. | n.a. | n.a. | n.a. |
| Disease progression - improving | yes | n.a. | yes | n.a. | n.a. | n.a. | yes | n.a. | n.a. | n.a. | n.a. |

The following paragraphs provide brief case summaries of patients A-G. Single case reports of patient H and I have recently been published [12, 13].

Patient A

At the age of 3.5 years, patient A was admitted to a children’s hospital with cough for 3 weeks and increasing food refusal. Prior to admittance, she also developed tachypnea, prolonged cough attacks, and perioral cyanosis. She presented in reduced general condition and poor nutritional status (height and weight for age <1^st^ percentile acc. to CDC growth charts[19]) with tachydyspnea, global respiratory insufficiency (pCO_2_ 46.9, pO_2_ 51.7 mmHg), an oxygen saturation of 85% in room air, and desaturations <50% during cough attacks. There was no fever or other clinical signs of infection. According to her medical history, she had suffered from prolonged H1N1 pneumonia 3 months prior to admission, and she also had a history of a severe upper respiratory tract infection at the age of 3 months, which had been treated with antibiotics. She is a former moderately preterm infant born at 36+4 weeks gestational age via secondary Caesarean section due to premature placental detachment. There were no postnatal complications. Her parents are healthy, and her older brother and sister never had any medical problems. First, the patient was admitted with suspected pneumonia. As there was no improvement despite intravenous antibiotic treatment, a CT thorax scan and a bronchoscopy were performed. The bronchoalveolar lavage fluid (BALF) showed a milky appearance, and, together with the CT scan findings, alveolar proteinosis was suspected. She was referred to a specialist center, where whole lung lavage therapy (WLL) was initiated and genetic diagnostic was arranged. In a second step, a lung biopsy was taken, and the diagnosis was confirmed. Genetic testing revealed that she was the homozygous carrier of a novel p.Arg199X nonsense mutation. In two years of clinical follow-up, the patient underwent 5 WLL and is clinically stable, receiving continuous supplemental oxygen. Future WLL will be done if deterioration is noted. A percutaneous endoscopic gastrostomy tube placed at the time of diagnosis could be removed after one year of constant weight gain (weight for age 31^st^ percentile).

Patient B

Patient B presented at the age of 6 years with cough, dyspnea, and chronic tachypnea. She had no relevant medical history. At the time of presentation, she was hypoxic in room air, and her height and weight for age were both below the 5^th^ percentile. Two segmental lung lavages were performed, and, as infection was ruled out, the diagnosis of PAP was suspected. She was negative for GM-CSF autoantibodies and showed elevated serum levels of GM-CSF. Genetic testing revealed that she carried a novel c.1125+1G>A donor splice site mutation at the exon 12/intron 12 border in homozygosity. In 2,5 years of follow-up, the girl underwent three WLL and showed improvement. Her best respiratory status is given as asymptomatic. Her height and weight for age increased to the 10^th^ (height) and 75^th^ (weight) percentile, respectively. Future WLL therapy will be initiated as needed.

Patients C and D

Sisters C and D also have a consanguineous family background. Patient C was presented at the age of 2 months, when her parents noted dyspnea on exertion and poor weight gain (weight for age 10^th^ percentile). She was a term-born infant with no relevant family, antenatal, birth, or neonatal history. As there were no signs of infection except nonspecific opacifications in both lungs on the chest film, extensive diagnostics were performed including a CT chest scan and a lung biopsy, which showed a periodic acid-Schiff (PAS)-positive stain. During a follow-up time of 7 years, she developed a transient need for oxygen supplementation, bronchiectasis, hepatomegaly, clubbing, and a moderate restrictive pattern in the lung function tests. She underwent 5 WLL and showed good clinical improvement during the last years. No further WLL is anticipated. Her weight for age is now on the 50^th^ percentile. Her sister, patient D, also was genetically investigated, when she developed chronic cough at the age of 19. She had no relevant medical history and showed normal growth. Lung function was normal, and there were no other clinical signs for a chronic lung disease. Her genetic test came back positive for the same homozygous duplication of *CSF2RA* exon 8. Both parents are heterozygous carriers of this mutation. As there was no worsening of symptoms, extensive and invasive diagnostic tests as well as WLL therapy were not indicated for this patient.

Patient E

The boy was first presented aged 4 ½ years with acute onset of dyspnea and cough. He had a history of recurrent pneumonia, mild mental impairment, and failure to thrive (height and weight for age 10^th^ percentile, respectively). Birth and neonatal history were normal, and there was no relevant family history. He was admitted with acute respiratory failure and required invasive ventilation. Extensive diagnostic tests did not reveal any cause of respiratory distress, until BALF analysis showed evidence of PAP. A lung biopsy also was consistent with the diagnosis. Genetic analysis revealed that he was the homozygous carrier of a deletion of exons 2-13 of the *CSF2RA* gene. In 9 years of follow-up, the boy received 24 WLL. His current respiratory status is given as symptomatic and progression is noted. While his height for age increased to the 90^th^ percentile, the boy is still underweight with a weight for age below the 5^th^ percentile. Future WLL therapies will be performed as needed.

Patient F

The girl was first presented at the age of 8 years. She is the third child in her family. In early pregnancy, intrauterine fetal death and abort of her twin occurred. Furthermore, her mother reported another abort in early pregnancy as well as two normal pregnancies from which two older brothers emerged. The 4-year-older brother is healthy; while the 3 year older brother has a history of Crigler-Najjar syndrome type 2 and mild eosinophilic colitis. The mother is the daughter of the father’s cousin. The mother’s grandmother, who is also the aunt of the father, has thirteen siblings of which some died in early childhood. The patient’s mother has 8 siblings of whom some are married to siblings of the father, and several children in this lineage have mental disabilities. One of these children is a girl, who was diagnosed with an interstitial lung disease of uncertain origin at the age of 3 years.

The patient had susceptibility for infections since early childhood with at least 8 feverish respiratory tract infections per year. At the age of 7 years, she was admitted for the first time to a hospital with pneumonia, which was treated with antibiotics. One year later, she was again admitted with complicated *Mycoplasma pneumoniae* infection. Chest radiographs showed markedly pronounced bilateral infiltrates. Further diagnostics were performed including a chest CT scan that showed a nonspecific interstitial pneumonia with distinct inflammation and bronchiectasis. BALF analysis revealed neutrophil granulocytosis, an increased number of mast cells, and a markedly increased number of PAS stain-positive cells, and was therefore suspected to suffer from PAP. She was referred to our Department for further diagnostics. A lung biopsy showed massive deposits of granular and myelin-like layered surfactant protein A and type II pneumocytes. Genetic analysis confirmed the diagnosis. She had a homozygous deletion of *CSF2RA* exons 2-13. Because of her good general condition and no respiratory symptoms, there was no indication for WLL treatment so far.

Patient G

Patient G developed dyspnea and fever at the age of 3 years. A chest x-ray showed diffuse bilateral infiltrates, and he was started on antibiotics. As no improvement occurred after 14 days, he was referred to a pneumology specialist center for adults, where the diagnosis of PAP was presumed and a WLL therapy was suggested. Open lung biopsy was performed, showing thickened alveolar septa in addition to typical findings suggestive of PAP. Hereditary PAP was suspected for several years, because GM-CSF antibodies in serum and BALF were negative on several occasions and other primary and secondary forms were ruled out. However, the first genetic analysis did not reveal any mutation. Ten years later, multiple ligation-dependent probe amplification (MLPA; MRC Holland, The Netherlands) demonstrated that he was a homozygous carrier of a large deletion, removing the complete *CSF2RA* gene locus as well as flanking sequences on the Xp22.3 and Y11.3 chromosomes. Both parents were heterozygous carrier of this defect. During the following 13 years, he received 25 unilateral and 12 whole lung lavages. Over the course of time, he remained clinically stable, though he showed failure to thrive with a body weight constantly below the 10^th^ percentile. To date, at the age of 15 years, he has a normal oxygen saturation in room air (96%), but is hypoxic on exertion (85% after 3 min. cycling test, 52 watt). His weight is slowly improving. Further WLL are anticipated.

Patient H[12]

“The patient was the 2^nd^ of 3 living children; born at term with no immediate postnatal respiratory distress. The family history was unremarkable for pulmonary or other rare diseases; the parents were consanguineous and of Turkish descent. At age 2 ½ years during an acute respiratory tract infection with productive cough and fever with no response to antibiotics, intermittent cyanosis occurred and the child was referred to our centre because of chronic tachypnoea and weight loss. Because of a typical chest [..] CT and chest x-ray […], BAL macroscopic appearance […] and microscopy […], and after exclusion of infectious or metabolic causes or malignancy, PAP was suspected and confirmed by histology […]. At presentation the child had global respiratory insufficiency [...] [, and] based on the clinical diagnosis of pulmonary proteinosis we initiated WLL [treatment]. [...] Because at clinical diagnosis of the patient, both the exact cause of [...] PAP and effective treatments in small children were unknown, empirical high dose glucocorticosteroids in pulses were used [...] and under the impression that they might be helpful, systemic corticosteroids were used for prolonged periods until the age of 7.5 years. During this time, azathioprine as a steroid saving agent was also used without any apparent benefit. However, we clearly observed that severe infectious complications were only observed during the time of increased immunosuppression by these agents [...]. At 5 years of age an Aspergillus fumigatus infection with formation of a cavity, leading to severe cardio-respiratory failure and resuscitation followed by resection of the left lower lobe [...]. At age 7.5 years she suffered a pulmonary para-influenza infection, leading to ARDS and necessitating mechanical ventilation. Additionally, the child had many mild respiratory exacerbations, mostly believed to be induced by viral upper- and lower respiratory tract infections. [...] Nutritional support was optimized with the help of a percutaneous gastrostomy [..] placed at the age of 3 years, which was used regularly; the gastrostomy was changed to a jejunostoma at the age of 6 ½ years. [...] Empirically, we found that WLL were the most efficient treatment. This was clearly shown for the short term; during 49 instances investigated until the age of 11, the amount of nasal oxygen flow was reduced in 40 after the lavages [...]. This effect could be sustained for many years demonstrating long term efficacy. Up to the age of 10 years,WLL were done more or less to ameliorate partial respiratory insufficiency, i.e. to decrease the need for additional oxygen. From year 10 onward, we performed one lavage per month, in order to try to completely clear the lung from its proteinosis load. This approach was very successful and resulted in complete resolution of partial respiratory insufficiency for the first time. The patient started puberty, growth and weight were sustained by oral nutrition without [further] need of using the percutaneous tube and the dependency on supplemental oxygen up to that point in time, could be finished. This also led to increased self-confidence and better integration at school. Also, the lung function improved very rapidly and chest radiograph cleared to almost normal.“

Patient I[13]

“[The patient is ] Three-year-old girl, the youngest of three female siblings born from consanguineous (first cousins) parents of Moroccan ancestry. Her personal and familial history were otherwise uneventful, and she had had normal growth with weight and height the 35th and 75th percentiles. She presented in September 2010 with six day fever, coughing, nasal discharge and hypoxemia (TcSaO2 93% breathing room-air). All remaining family members were symptom-free. A chest X-ray showed right upper lobe […] consolidation and a bilateral interstitial pattern […].Serum antibodies to Chlamydia, Coxiella and M. pneumoniae were all negative […].She was administered oral azithromycin and received oxygen supply for three days and was discharged 5 days after admission. Three weeks later clinical worsening with increasing cough, breathlessness and hypoxemia (TcSaO293% on room air) were noted. A chest X-ray showed a bilateral reticulonodular pattern without […] consolidation […]. She was treated with antibiotics and antituberculosis drugs until work-up for Mycobacteria proved to be negative. On bronchoalveolar lavage (BAL) a serum-like liquid with abundant macrophages and scarce neutrophils was recovered. Serum antibody titer to M. pneumoniae was positive (equal or higher than 1/1280). [A few weeks later] she was admitted again with polypnea 64/min, breathlessness on mild exertion and severe hypoxemia (TcSaO2 82%-85% on room air). She required oxygen supply 1-3 l/ min. Her weight had dropped to the 10th percentile, but her general condition was otherwise good. Her temperature was 37.2 [degrees celsius], she was pale, and had mild bilateral decreased breath sounds on chest examination. A chest CT-scan showed a striking bilateral “crazy-paving” pattern [...]. On BAL a milky liquid [...] with granular proteinaceous periodic acid-Schiff (PAS) positive material was recovered, supporting the diagnosis of PAP which was confirmed by surgical pulmonary biopsy [...]. Molecular genetics investigation [...] showed blood leukocyte abnormalities suggesting defective GM-CSF receptor: no GM-CSF dependent phosphorylation of STAT5, increased serum GM-CSF concentration (73.66 pcg/ml), undetectable GM-CSF receptor alpha-chain. The patient was found be[ing] homozygous for two mutations in the CSF2RA: c.50C > G (p.Ala17Gly) in exon 3, and c.586G > C (p.Gly196Arg) in exon 7. GM-CSF autoantibody testing was negative. [...] The investigation of the family showed that the parents and one of the patient’s sisters were heterozygous for both mutations, the STAT5 phosphorylation index was normal and GM-CSF receptor alpha and beta proteins were detected. The other sister aged 7 years was also found to be homozygous for both mutations [...]. GM-CSF dependent STAT5 phosphorylation and the GM-CSF receptor alphachain were undetectable. However she had had not significant symptoms, her physical examination was unremarkable and her TcSaO2 was 98% on room air. [...] [The patient] underwent [partial lung lavages] [...] with a 1-week interval between the right and left lung lavages [This procedure was repeated after 3 months.]. [...] She was asymptomatic 3 weeks after the last lavage with TcSaO2 98% breathing room air, and her chest-ray changes had improved.“ After the family moved to Germany, the girl was presented for regular WLL treatment at our centre. Until November 2013, aged 5 years, she underwent 34 WLL in 4-8-weekly intervals. This approach was very successful and resulted in complete resolution of partial respiratory insufficiency. Still, her weight-for-age was < 10th percentile, and nutritional support was optimized with the help of a percutaneous gastrostomy placed at the age of 5 years.
