# Supplementary figures and images for: Characterization of CSF2RA mutation related juvenile pulmonary alveolar proteinosis
Source: Orphanet J Rare Dis. 2014 Nov 26;9:171. doi: 10.1186/s13023-014-0171-z (PMC4254258; doi:10.1186/s13023-014-0171-z)

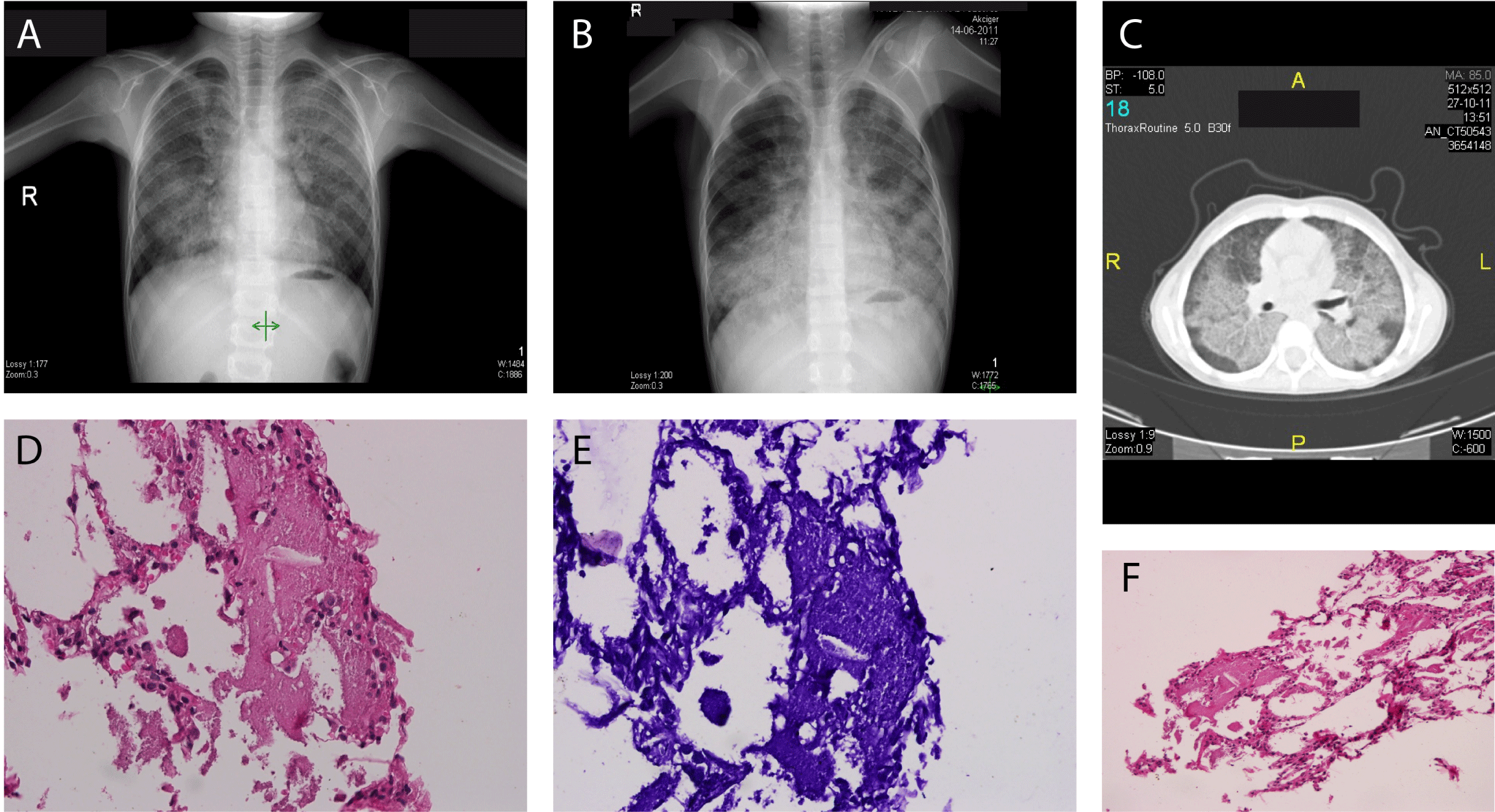

Supplement: Additional file 2: Figure S3. — Radiological and histopathological findings in juvenile PAP. A./B. Bilateral ground-glass opacifications and infiltrates as commonly seen in PAP (A. male, 9y; B. female, 5y). C. CT chest scan displaying a crazy paving pattern (female, 6y). D-F. Lung biopsy tissue shows PAS positive granular material in the alveoli. [file 13023_2014_171_MOESM2_ESM.gif]

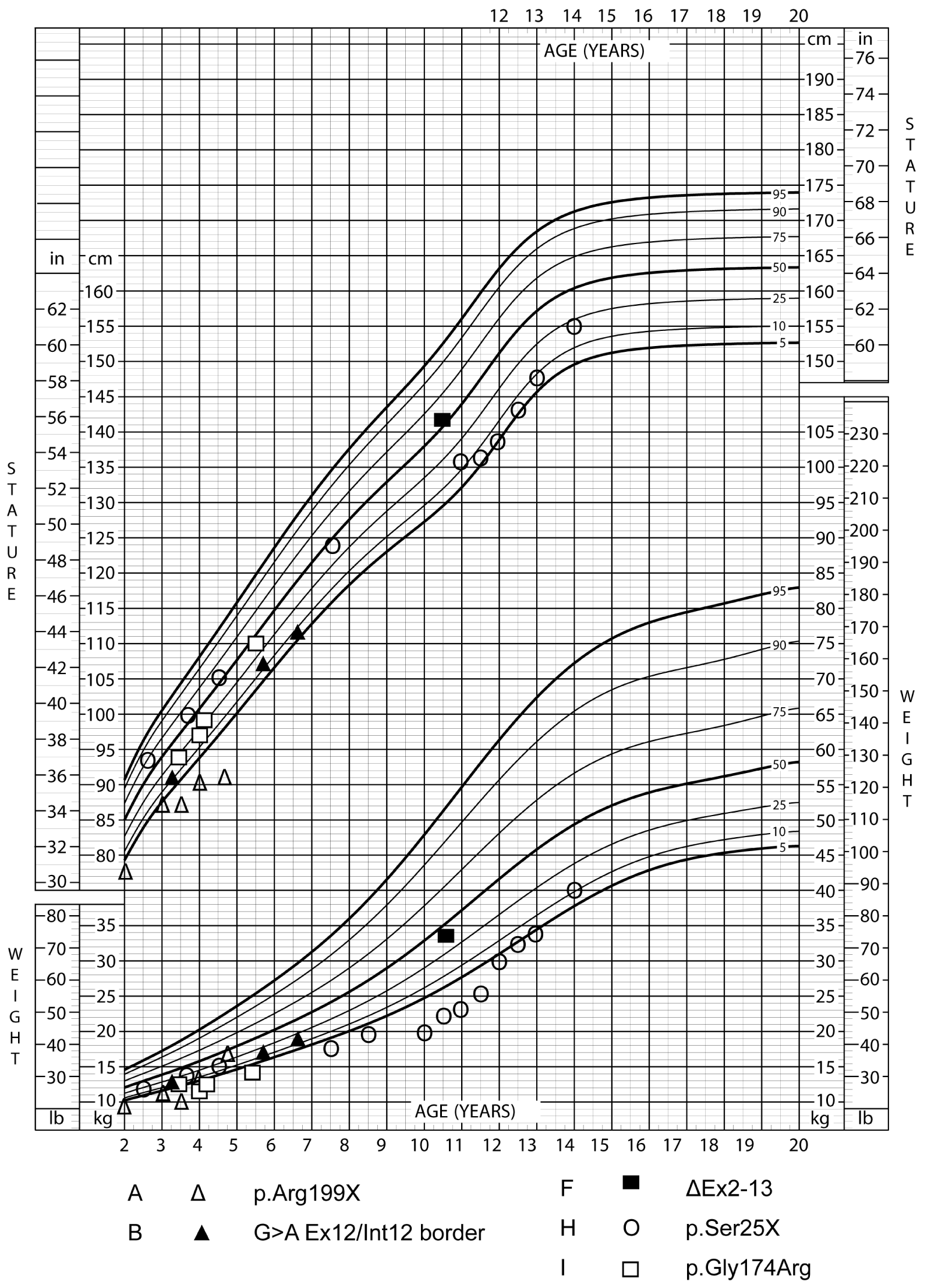

Supplement: Additional file 3: Figure S1. — Growth charts of female PAP patients. Stature-for-age and weight-for-age development of female PAP patients displayed on CDC growth charts [22]. Mostly, weight-for-age development fails to reach the 50th percentile. Of note, patient H showed marked improvement of physical development after initiation of regular WLL treatment at the age of 10 years. [file 13023_2014_171_MOESM3_ESM.gif]

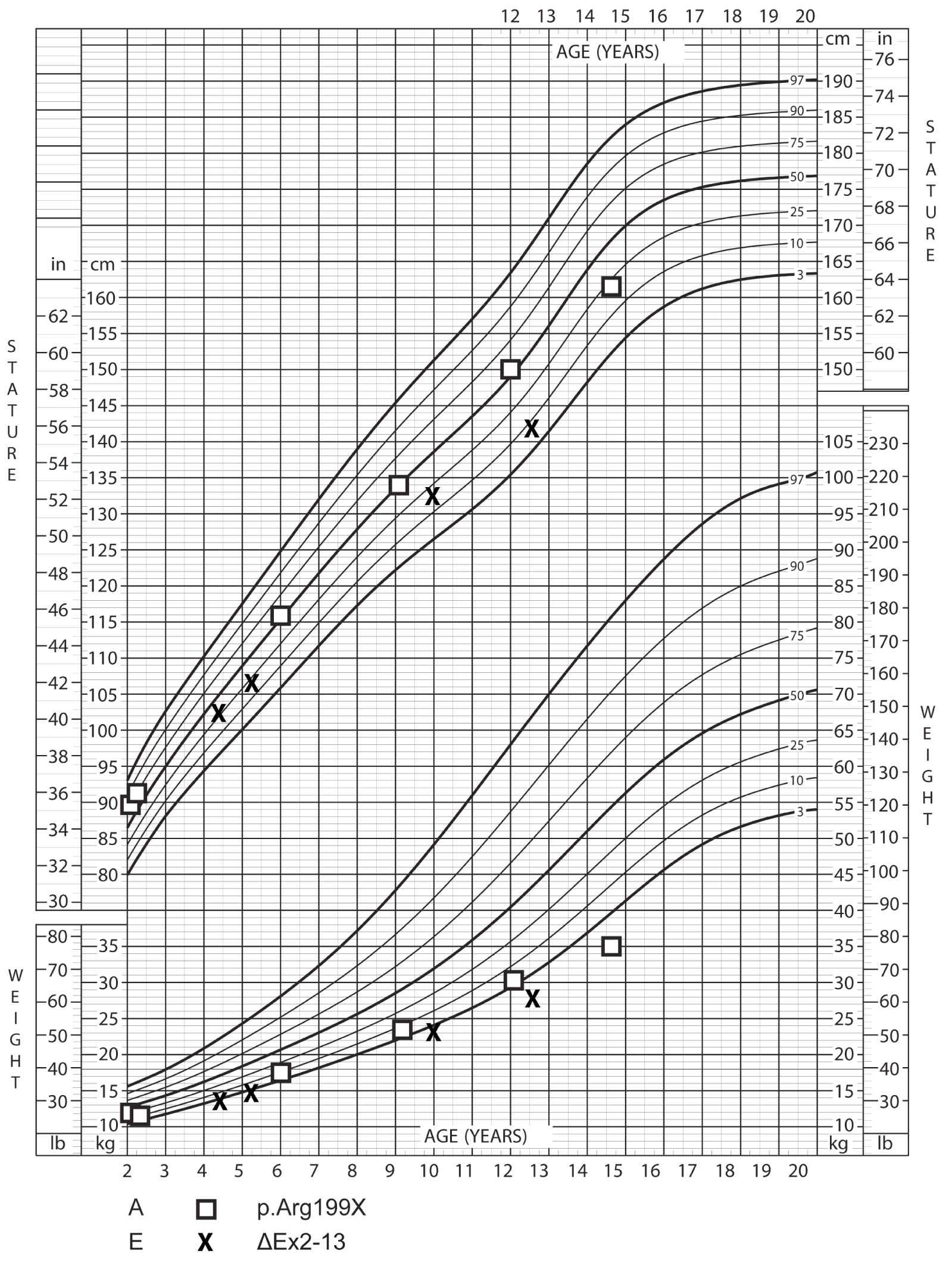

Supplement: Additional file 4: Figure S2. — Growth charts of male PAP patients. Stature-for-age and weight-for-age development of male PAP patients displayed in CDC growth charts [22]. In general, especially the weight-for-age parameters show a constant development below the 10th percentile, exceeding the developmental deficits seen in female patients. [file 13023_2014_171_MOESM4_ESM.gif]
